# Predicting clinical outcome in patients undergoing VA-ECMO

**DOI:** 10.1186/s13054-019-2350-8

**Published:** 2019-02-14

**Authors:** Antoine Kimmoun, Bruno Levy

**Affiliations:** 1 Service de Médecine Intensive et Réanimation Brabois, CHRU de Nancy, 54511 Vandœuvre-lès-Nancy, France; 2 U1116, Défaillance Circulatoire Aigue et Chronique, Faculté de Médecine de Nancy, 54500 Vandœuvre-lès-Nancy, France; 3 Université de Lorraine, CS25233, 54052 Nancy cedex, France

Cardiogenic shock is defined by a myocardial failure responsible for low cardiac output and tissue hypoperfusion despite an adequate intravascular volume status. From this non-specific definition, cardiogenic shock can in fact have many degrees of severity ranging from pre-shock to refractory shock states. Refractory cardiogenic shock is generally defined as cardiogenic shock with sustained hypotension, end-organ hypoperfusion, and hyperlactatemia despite adequate intravascular volume and high-dose inotrope and vasopressor support [1]. In such situation, the VA-ECMO device aims to recover both circulatory output and tissue perfusion at an early stage in order to prevent the irreversible consequences of the organ hypoperfusion state [2]. Thus, despite limited evidence, VA-ECMO is increasingly used for the management of refractory cardiogenic shock (RCS) while awaiting myocardial recovery or the bridge to long-term assistance or transplantation [3]. However, despite technological advances and better quality of care, mortality still remains at an unacceptable high rate at nearly 50% [4]. Furthermore, even in survivors, long-term health-related quality of life parameters are also impaired [5]. Thus, identification of patients in whom such therapy may or may not be beneficial is essential in order to improve patient selection but also so as not to discredit a humanely and financially costly, albeit potentially useful technique. For this purpose, many teams have developed predictive scores (PSs) for RCS patients under VA-ECMO.

## General principles for the development of a predictive score

Aside from complex statistical considerations, it is imperative to remind certain key facts regarding prediction models. First, the development of a PS, which is one sub-category of prediction models, should follow the TRIPOD checklist (see www.tripod-statement.org) [6]. Second, a prediction model should be developed on a large (meaning multicentric) specific population (ideally prospective) with a clearly defined outcome (most often short-term mortality for patients under VA-ECMO). Third, the cornerstone of the statistical analysis is most often (for short-term mortality) the multivariable logistic regression model on which the independent predictive variables are defined. The number of variables included in the model is contingent on numerous rules including, among others, the number of events (usually 1 variable for 8 to 10 events) in order to avoid overfitting. Fourth, model performance should be provided with both internal and external validation which, most of the time, represents a genuine challenge for this criterion. Finally, the interpretation of a PS, i.e., its potential value at bedside and/or its eventual implications in the health system, should be cautiously reported. From these general considerations, it is clear that the development of a PS under VA-ECMO is a complex and challenging process.

## Predictive score characteristics for patients under VA-ECMO: from general to specific scores

The various score characteristics are depicted in Table 1. The first PS was developed by Schmidt et al. in 2015 and aimed to predict in-hospital survival in patients under VA-ECMO for RCS [7]. For the development of this PS, the authors used a large retrospective multinational cohort of 3846 patients in which 12 variables were extracted to create the Survival After Veno-Arterial-ECMO (SAVE) score. This PS, with proper discriminative and good calibration performances, was the only PS validated on an external cohort. Although constructed on a strong methodology, namely large multicentric population, validation cohort, and very good dissemination with a ready-to-use online calculator, this PS also suffered from several limitations. For instance, being based on a registry, the percentage of missing data was high and certain relevant biomarkers such as lactate or troponin were not included. Above all, this general PS did not address specific populations such as patients in cardiac arrest. Thus, thereafter, some authors endeavored to improve this PS by combining lactate at ECMO initiation to the SAVE score [8]. Using a method similar to Schmidt et al., others also addressed specific etiologies. For example, Muller et al. and more recently Wang et al. developed PSs for specific populations: respectively acute myocardial infarction and post-coronary artery bypass grafting [9, 10]. The major pitfalls of these two PSs were related to first, the population which was small, mono- or bicentric, and second, the absence of an external validation cohort. Both severely limited the generalization and dissemination of the results into clinical practice.

**Table 1:** Summary of the main predictive scores developed for patients under VA-ECMO for refractory cardiogenic shock

Study	Year	Population	Sample size	Outcome prediction	Number of items retained/number of deaths	Internal Validation	AUROC	External validation
Schmidt et al. [7]	2015	General RCS multicentric	3846	In-hospital survival	12/2245	Yes	0.68 [95% CI 0.66–0.69]	Yes AUROC 0.90 [95% CI 0.85–0.95]
Muller et al. [9]	2016	Post AMI RCS bicentric	138	In-hospital survival	7/65	Yes	0.84 (95% CI 0.77–0.91	No
Wang et al. [10]	2018	Post CABG monocentric	166	In-ICU mortality	6/92	Yes	0.85 (95% CI 0.79–0.91)	No

*AUROC* area under receiver operating characteristics, *RCS* refractory cardiogenic shock, *AMI* acute myocardial Infarction, *CABG* coronary artery bypass grafting

## What are predictive scores for?

While the development of a PS is a complex process, the interpretation and “operating instruction” of such PSs for clinicians is even more challenging. Indeed, clinicians are tempted to use PSs at bedside for the wrong reasons. All of these scores were designed to predict outcome in patients who are already under VA-ECMO. Thus, they are not intended to determine whether an individual patient should be cannulated. The CRASH score (Catecholamine Refractoriness and Assistance guide based on cardiogenic Shock Hemodynamics), with its many limitations due to the methodology, was designed for this purpose but was never externally validated [11]. Furthermore, as mentioned by Schmidt et al. in their study, extreme SAVE scores ≤ 10 still featured an in-hospital survival of 18% [7]. Moreover, these PSs were all designed, as mentioned above, at a given time for a specific population. Rapid changes in practices in the field of RCS and VA-ECMO are expected and a PS may very quickly become obsolete. Thus, these PSs should be regularly updated. Finally, these PSs are probably more suitable for larger-scale assessment between periods or centers. They could also be used for patient stratifications in clinical research [7].

In summary, PS for patients under VA-ECMO for RCS could help the physician at bedside by providing information on outcome prediction as well as be of interest for research purposes. In all instances, we believe that they should never be used as a decision tool to indicate or to limit access to VA-ECMO.
